# Prognostic significance of heparanase expression in primary and metastatic breast carcinoma

**DOI:** 10.18632/oncotarget.23560

**Published:** 2017-12-21

**Authors:** Olga Vornicova, Inna Naroditsky, Ilanit Boyango, Shlomit S. Shachar, Tanya Mashiach, Neta Ilan, Israel Vlodavsky, Gil Bar-Sela

**Affiliations:** ^1^ Division of Oncology, Rambam Health Care Campus, Haifa, Israel; ^2^ Departments of Pathology, Rambam Health Care Campus, Haifa, Israel; ^3^ Cancer and Vascular Biology Research Center, Bruce Rappaport Faculty of Medicine, Technion-Israel Institute of Technology, Haifa, Israel; ^4^ Statistics, Rambam Health Care Campus, Haifa, Israel

**Keywords:** heparanase, breast carcinoma, metastasis, survival, discordance

## Abstract

High levels of heparanase are detected in many types of tumors, associated with bad prognosis. Typically, heparanase levels are evaluated in a biopsy taken from the primary lesion, whereas its expression by the resulting metastases is most often unresolved. This becomes critically important as anti-heparanase compounds enter advanced clinical trials. Here, we examined the expression of heparanase in pairs of primary and the resulting distant metastases of breast carcinoma. Interestingly, we found that heparanase expression in the metastatic lesion does not always reflect its expression in the primary tumor. Accordingly, in some cases, the primary lesion was stained positive for heparanase while the metastasis stained negative, and vice versa. Heparanase discordance occurred in 38% of the patients, higher than that reported for hormone receptors, and was associated with bad prognosis. Thus, examination of heparanase levels in the tumor metastases should be evaluated for most efficient precision medicine applying heparanase inhibitors. Furthermore, we found that in stage I breast cancer patients strong heparanase staining is associated with shorter overall survival. Thus, heparanase levels can assist in the diagnosis and in determining the necessity and type of treatment in early stage breast cancer.

## INTRODUCTION

Heparanase is an endo-β-D-glucuronidase that cleaves heparan sulfate (HS) side chains of heparan sulfate proteoglycans (HSPG). This activity is responsible for remodeling of the extracellular matrix (ECM), thereby promoting cell dissemination associated with tumor metastasis, angiogenesis and inflammation [1, 2]. Heparanase expression is low in normal epithelia but its expression is up-regulated in many carcinomas as well as sarcomas and hematological malignancies [1–4]. Notably, cancer patients exhibiting high levels of heparanase had a significantly shorter postoperative survival time than patients whose tumors exhibit low levels of heparanase, thus supporting its pro-metastatic function [1, 2]. More recent studies provided compelling evidence that tie heparanase levels with all steps of tumor formation including tumor initiation, growth, metastasis, and chemoresistance [5–11]. These and other results indicate that heparanase is causally involved in cancer progression and hence is a valid target for anti-cancer drug development. This notion is reinforced by preclinical studies revealing a marked inhibition of tumor growth in mice treated with heparanase-inhibitors, now in phase I/II clinical trials in cancer patients [12–14].

The same principles are also relevant to breast cancer. Heparanase is undetected in normal breast epithelium but its expression is induced in human breast carcinoma, associated with increased tumor metastasis and larger tumor size [15–17]. Similarly, overexpression of heparanase promotes, while anti-heparanase siRNA decreases the growth, angiogenesis, and metastasis of breast carcinoma cells [18–20]. In addition, heparanase inhibitors were found to efficiently attenuate the tumorigenic capacity of breast carcinoma cells [21, 22], altogether implying that heparanase plays a decisive role in breast cancer [17].

Most often, anticancer treatment is initiated after resection of the primary tumor and is directed against remaining tumor cells and micrometastases. Paradoxically, however, most studies examined heparanase levels in the primary tumor and not in the resulting metastases that are the prime target of heparanase inhibitors. This becomes critically important as anti-heparanase compounds enter advanced clinical trials [23]. Recently, we reported that most melanoma metastases are stained positive for heparanase [24]. Moreover, we found that in stage IVc melanoma patients, high heparanase expression in the metastases predicts poorer prognosis, clearly implying that heparanase levels in the metastatic lesions affect the disease outcome [24]. Here, we examined the expression of heparanase in primary and metastatic breast carcinoma. Unlike the melanoma cohort, in this study, we obtained pairs of the primary and the resulting distant metastases from the same patient. Interestingly, we found that heparanase expression in the metastatic lesion does not always reflect its expression in the primary tumor. Accordingly, in some cases, the primary lesion was stained positive for heparanase while the metastasis stained negative, and vice versa. Notably, the cases in which heparanase expression was changed had a poorer prognosis compared with cases in which heparanase expression was not altered. Furthermore, we found that in stage I breast cancer patients strong heparanase staining is associated with shorter overall survival. Thus, heparanase levels can assist in the diagnosis, necessity, and type of treatment in early stage breast cancer.

## RESULTS

### Heparanase staining is different in metastases vs primary tumor

**Figure 1:**
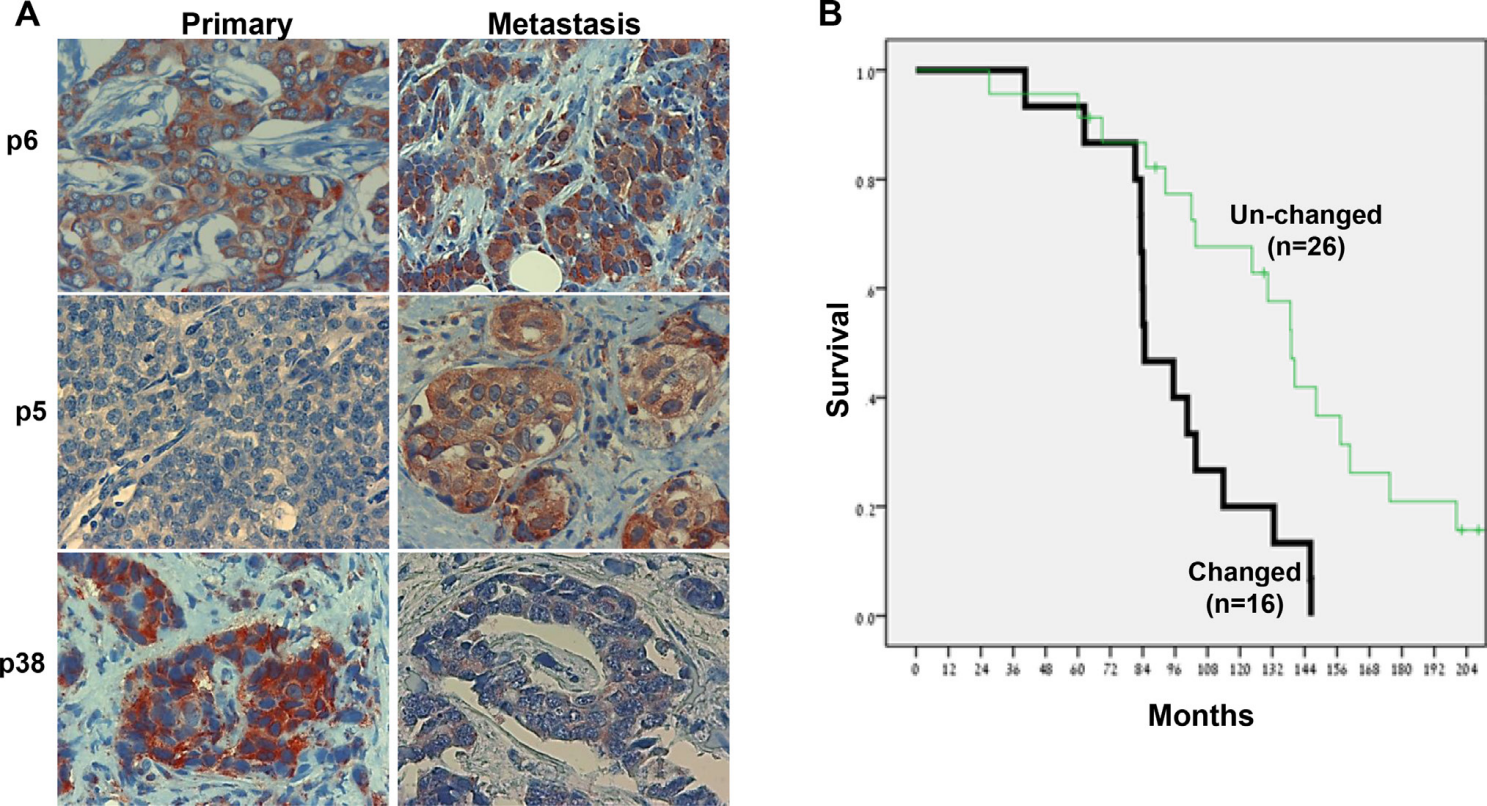
Discordance of heparanase staining in primary vs metastatic breast cancer (**A**) Immunostaining. Forty-two pairs of primary breast carcinomas and resulting metastases were subjected to immunostaining applying anti-heparanase antibody. Shown are representative photomicrographs of cases in which heparanase staining appeared comparable in the primary and metastatic lesions (p6; upper panels), cases in which heparanase was low in the tumor cells of the primary lesion but appears high in the metastases (p5; second panels), and cases in which heparanase staining was strong in the primary tumor but negative in the resulting metastases (p38; third panel). (**B**) Kaplan-Meier survival analysis. The survival of patients in which heparanase staining in the primary and metastases appeared similar (Un-changed; *n* = 26) was compared to patients in which heparanase staining was changed in the primary vs metastases (Changed; *n* = 16). Note that discordance of heparanase expression correlates with a significant decrease in patient survival (HR 0.035; *p* = 0.005). Original magnifications: × 100.

In order to examine the expression of heparanase in primary tumors vs metastases, we collected specimens from 121 breast carcinomas. Fifty patients developed recurrent disease during follow-up, but paraffin slides were available only from 42 patients. Applying immunohistochemistry we found that in some cases, the primary tumor and resulting metastases exhibit similar staining pattern of heparanase, as exemplified in patients 6 (p6; Figure 1A, upper panel) 58, 8, 9, 10, 27 & 28 (Supplementary Figure 1). In other cases, however, we found that heparanase staining is changed significantly in the primary tumor vs its metastasis, in both ways. As exemplified in patients 5 (p5; Figure 1A), 57 and 25 (Supplementary Figure 2), the primary tumor is stained negative for heparanase while the metastasis lesion is stained strongly. The opposite scenario occurs in patients 38 (p38; Figure 1A, third panel) and 56 (Supplementary Figure 2, middle panels) where the primary tumor is stained strongly whereas the metastasis is stained negative for heparanase. Staining extent was typically high (i.e., 70–80% of the cells are stained positive for heparanase), and this parameter was not included in subsequent analyses.

When combining heparanase staining in the primary tumor and its metastases (42 pairs), the following results were obtained: In 26 cases there was no difference between heparanase staining in the primary tumor vs metastases (strong remained strong, weak or negative remained weak or negative), whereas in 16 cases (38%) heparanase expression was changed significantly (Table 1). Interestingly, patients (16) in which heparanase staining between primary tumor and metastasis was changed exhibited worse prognosis vs patients exhibiting stable heparanase pattern (HR 0.035; *p* = 0.005) (Figure 1B).

**Table 1:** Heparanase staining in primary and metastatic breast cancer (42 patients)

	Number of patients (%)
Similar:	26 (62)
Primary weak/ metastasis weak (−/−)	14
Primary strong/ metastasis strong (+/+)	12
	
Not similar:	16 (38)
Primary weak/ metastasis strong (−/+)	10
Primary strong/ metastasis weak (+/−)	6

### High heparanase levels predict bad prognosis in stage I breast cancer patients

**Figure 2:**
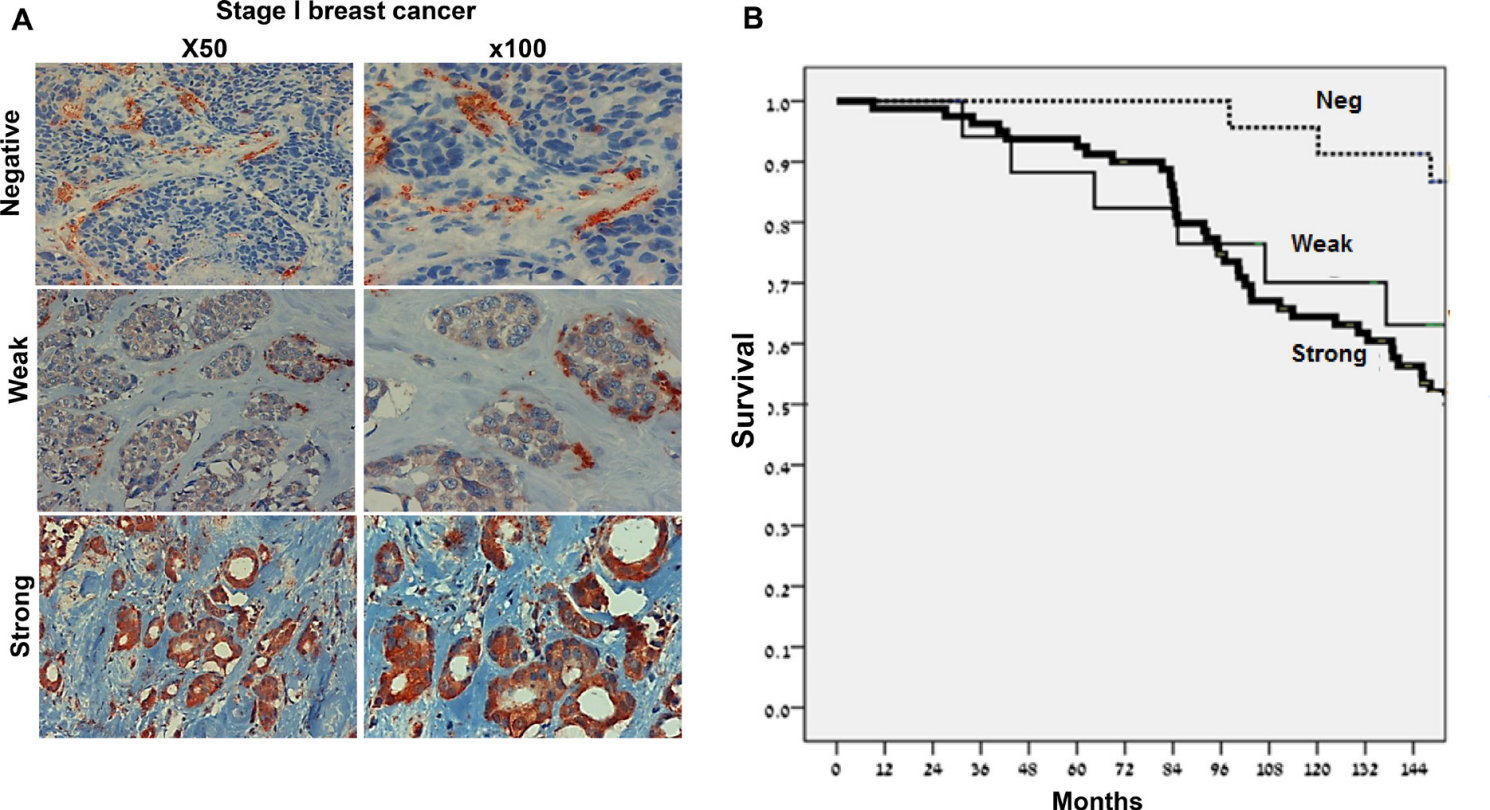
High levels of heparanase are associated with reduced survival of stage I breast cancer patients (**A**) Immunostaining. Stage I breast carcinomas were subjected to immunostaining applying anti-heparanase antibody. Shown are representative photomicrographs of cases exhibiting no staining of heparanase in the tumor cells (positive staining is detected in stromal cells; upper panels), weak (middle panels) or strong (lower panels) staining. (**B**) Kaplan-Meier survival analysis. The survival of patients was examined according to heparanase staining intensity. Note bad prognosis of stage I breast cancer patients that exhibit strong staining of heparanase (HR 4.52; *p* = 0.03).

We next analyzed the staining of heparanase in the primary tumors in relation to clinical parameters. Demographic and clinical description of the patients is summarized in Table 2. The median age for the entire group was 53. One-third of the patients (35%) were diagnosed with stage I disease (Ia-Ib), 43% were diagnosed with stage II disease, and 23% with stage III. Fifty-nine patients (49%) had lymph node involvement at presentation (Table 2). The extent of heparanase staining appeared similar in most specimens. In contrast, staining intensity varied considerably among patients. Of the 121 biopsies of primary breast carcinoma, 51 exhibited strong staining of heparanase (+2; Figure 2A, lower panels), of which 22 (43%) were diagnosed later with metastatic disease. Seventy samples were stained negative (Figure 2A, upper panel) or weak (+1; Figure 2A, middle panels) for heparanase, of which 28 (40%) developed metastatic disease (Table 3). There were no statistically significant differences between the groups. Further statistical analysis of the clinical data revealed the prognostic significance of tumor stage, number of involved lymph nodes, and the status of estrogen receptor (Table 2), as expected. This implies that our patient cohort exhibits typical characteristics of breast cancer. Strong heparanase staining in the primary tumor of the entire cohort was not a prognostic factor for metastatic disease. However, multivariate subgroup analysis showed that in stage I patients, negative staining for heparanase is associated with significantly better disease-free survival (HR 4.52; *p* = 0.03) (Figure 2B).

**Table 2:** Demographic and clinical characteristics of the patients enrolled in this study

		*N* (%)	No of events^*^ (%)	*P* value^**^	HR	CI
Stage	IA or IB	41 (34)	12 (29)	0.008	1.00	
.	IIA	29 (24)	18 (62)	0.007	2.74	1.32-5.69
.	IIB	23 (19)	11 (48)	0.056	2.23	0.98-5.10
.	IIIA	19 (16)	12 (63)	0.002	3.55	1.57-8.03
.	IIIB	8 (7)	6 (75)	0.0001	5.97	2.20-16.20
.	DCIS (Tis)	1 (1)	1	0.399	2.41	0.31-18.74
Lymph nodes	None	60 (51)	24 (40)	0.013	1.00	
.	1-3	43 (36)	24 (56)	0.057	1.74	0.99-3.09
.	4+	15 (13)	11 (73)	0.005	2.87	1.38-5.96
.	Missing	3		.	.	.
Grade	I	8 (7)	3 (37)	0.803	1.00	
.	II	59 (53)	29 (49)	0.556	1.43	0.44-4.70
.	III	45 (40)	21 (47)	0.507	1.51	0.45-5.05
.	Missing	9		.	.	.
Estrogen receptors	Negative	27 (23)	18 (67)	.	1.00	.
.	Positive	91 (77)	41 (45)	0.028	0.54	0.31-0.93
	Missing	3				
HER-2	Negative	65 (81)	29 (46)	.	1.00	.
.	Positive	15 (19)	8 (53)	0.540	1.29	0.58-2.87
.	Missing	41		.	.	.
Progesterone receptor	Negative	42 (36)	22 (52)	0.92	0.718	
.	Positive	75 (64)	36 (48)		0.838	
.	Missing	4	.		.	

^*^Events = relapse or mortality ^**^p value is calculated for each group in relation to the other sub-groups.

**Table 3:** Heparanase staining intensity and recurrent disease in breast carcinomas

Heparanase staining intensity	Number of patients (%)	Recurrent disease (%)
Negative (0)	16 (13)	5 (31)
Weak (+1)	54 (45)	23 (42)
Strong (+2)	51 (42)	22 (43)

## DISCUSSION

Breast cancer is the most common type of malignancy in females; it is estimated that 246,660 new cases will be diagnosed in 2017 in the USA [25]. Despite noticeable progress in diagnostic and therapeutic approaches, many patients still develop metastases, resulting in about 40,000 deaths annually [25]. Treatment of metastatic breast cancer, as well as early-stage breast cancer, is based on the status of estrogen receptor (ER), progesterone receptor (PR), and epidermal growth factor receptor 2 (HER2). In addition, other factors are routinely used for therapy decision-making, including disease-free interval, site(s) of relapse, number of metastases, proliferation index and histological grade; Stage of disease remains the most important predictive factor [26].

Notably, phenotype discordance in hormone receptors (ER and PR) and HER2 status between primary and recurrent breast cancer has been repeatedly reported [27, 28] and accounts, at least in part, to treatment failure [29]. Moreover, studies employing gene expression profiling of primary breast cancers and matched axillary lymph node metastases have found that breast cancer metastases are molecularly distinct from their primary tumors, and many genes are differentially expressed between primary tumors and metastases [27]. Our results add heparanase to the growing list of proteins that are differentially expressed by the primary tumor and its metastases. The reason for this is not entirely clear, but may be attributable to the clonal nature of metastases and its genomic instability, leading to continuous alterations. According to this notion, metastatic disease evolution is associated with altering tumor biology. This concept is supported by a recent study showing that discordance continues also from the first to second metastases [30]. Interestingly, tumors in which heparanase expression was different between the primary tumor and its metastases exhibited inferior prognosis compared with tumors in which heparanase expression was stable (Figure 1B). This is in agreement with previous publications showing the same phenomena upon discordance in ER/PR/HER2 expression [27]. Reassessing the biological features of disease is not currently considered mandatory, but recent international guidelines recommend that when there is discordance of ER, PR, or HER2 between primary and metastatic tissues then their status in the metastases should be considered to direct therapy [31]. The same rationale should ideally be applied to anti-heparanase treatment, for most effective precision medicine. Clearly, the discordance in heparanase levels showed here needs to be confirmed in a larger cohort of patients, ideally employing also additional methodology such as quantitative PCR.

The role of heparanase in breast cancer has been extensively examined in preclinical studies, but clinical evidence is limited. A Recent publication, analyzing the results obtained in several independent patient cohorts, showed that high levels of heparanase are associated with poor 5-year survival in breast cancer patients [17]. Early, stage I, breast cancer is particularly challenging for treatment. According to accumulated data of tumor recurrence in early breast cancer patients, “triple negative tumors” have a more aggressive biological behavior than tumors expressing hormone receptor and/or HER2 [32]. Patients with hormone positive tumors usually receive adjuvant hormone therapy, but in this population, there is a group of patients that may have better chances of full recovery with the addition of chemotherapy to adjuvant treatment [33]. Recently, several multigene assays have been developed to improve patient selection among women with early stage, hormone positive and HER2 negative, breast cancer that have a higher risk for disease recurrence and may benefit from adding chemotherapy to adjuvant hormone therapy [34, 35].

Patients with hormone-negative tumors usually receive chemotherapy to reduce the risk of disease recurrence if their tumor was larger than 1 cm, and in certain cases even with only 6 mm tumor (NCCN Guidelines). Predictive factors for this group of patients that may reduce the need for chemotherapy are not known yet. Here, we found that high levels of heparanase in stage I breast cancer (with tumors smaller than 2 cm and without lymph node involvement) correlates with a 4.5-fold increased risk of disease recurrence (Figure 2B). This finding may serve as a new prognostic marker that may ease the debate of providing or not preventive chemotherapy for patients with stage I breast cancer. While this observation needs further confirmation in a larger patient cohort, it may turn important and beneficial in the clinic.

Taken together, the results clearly show that heparanase is critically important for the progression of stage I breast cancer. Moreover, we show for the first time the complexity of heparanase expression in the primary tumors vs metastatic lesions. Heparanase discordance occurred in 38% of the patients, higher than that reported for hormone receptors, and was associated with bad prognosis. Thus, examination of heparanase levels in the tumor metastases should be evaluated for most efficient precision medicine applying heparanase inhibitors.

## MATERIALS AND METHODS

### Study population

Paraffin blocks were obtained from 121 patients diagnosed with breast cancer (invasive ductal carcinoma) and treated in the Rambam Health Care Campus, Haifa, Israel, between the years 1990–2014. Fifty patients were diagnosed with recurrent metastatic disease during follow up, of which 42 had tissue samples available for immunostaining. These included metastases to lymph nodes (12), bones (8), liver (7), breast (6), lung (4), brain (1) and other organs. The other 71 patients were under surveillance for at least 10 years with no evidence of active disease. All patients received standard of care treatment for breast cancer, according to the time of diagnosis and were under surveillance in the Oncology Department, Rambam Health Care Campus, Haifa, Israel. Their performance was analyzed in correlation with pathological, demographic and clinical characteristics, including the stage of disease (TNM), pathological grade, estrogen and progesterone receptor status (ER, PR), HER2 expression (where available), metastatic disease, and treatment modality (chemotherapy, trastuzumab, hormonal and radiation therapies). Patients were excluded from final analyses if tissue samples were not available for staining. The study was approved by the Rambam hospital’s Helsinki Committee.

### Immunohistochemistry

Biopsies were subjected to immunostaining applying anti-heparanase antibody (#733) essentially as described [23] and staining of the entire specimen section was evaluated by a senior pathologist (IN) who was blind to clinical data of the patients. Staining was scored according to the intensity (0: none; +1: weak-moderate; +2: strong) and extent (i.e., percent of heparanase-positive cells) in the malignant cells. Specimens that were similarly stained with normal rabbit serum or by applying the above procedure but lacking the primary antibody yielded no detectable staining.

### Statistics

A comparison was made between the demographic data, the disease characteristics and the intensity of heparanase staining by using a bivariate logistic regression. Cox regression model was used to determine factors influencing survival, illustrated by Kaplan-Meier curves. The level of significance selected to examine the various parameters in this study was set at *p* ≤ 0.05. The data were processed using SPSS statistical software, version 21.0 (Chicago IL).

## SUPPLEMENTARY MATERIALS FIGURES


