# Supplementary material for: Prognostic significance of heparanase expression in primary and metastatic breast carcinoma
Source: Oncotarget. 2017 Dec 21;9(5):6238–44. doi: 10.18632/oncotarget.23560 (PMC5814208; doi:10.18632/oncotarget.23560)
Supplement: Supplementary file 1 [file oncotarget-09-6238-s001.pdf]

Prognostic significance of heparanase expression in primary and metastatic breast carcinoma

SUPPLEMENTARY MATERIALS

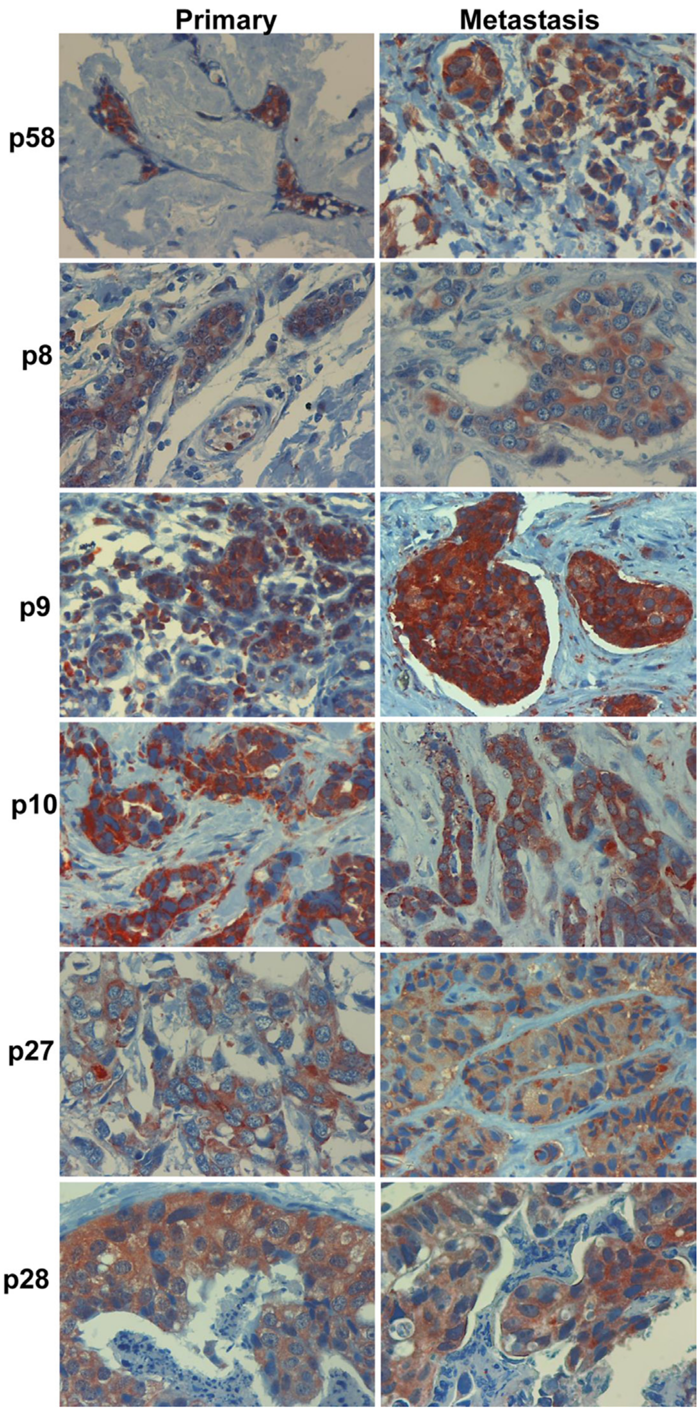

Supplementary Figure 1: Forty two pairs of primary breast carcinomas and resulting metastases were subjected to immunostaining applying anti-heparanase antibody. Shown are representative photomicrographs of patients (p) in which heparanase staining appeared comparable in the primary (left panels) and metastatic lesions (right panels).

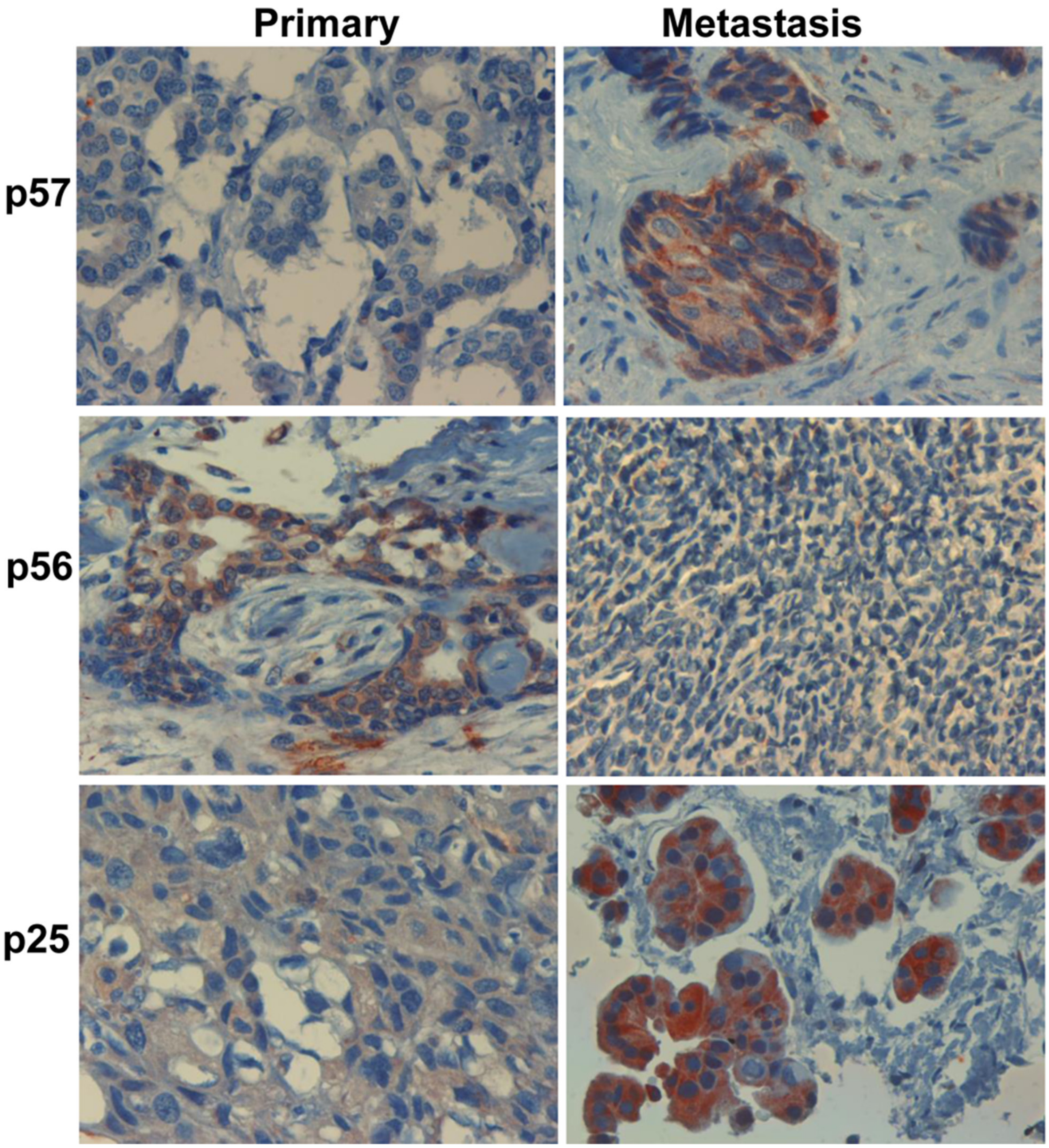

Supplementary Figure 2: Forty two pairs of primary breast carcinomas and resulting metastases were subjected to immunostaining applying anti-heparanase antibody. Shown are representative pairs of primary breast carcinoma and resulting metastases in which heparanase staining appears different. In patients 57 and 25 heparanase was low in the primary tumor but appears high in the metastases (p57, p25; upper and lower panels), whereas in patient 56 heparanase staining was strong in the primary tumor but negative in the resulting metastases (p56; middle panels).
